# Therapeutic education of patients with coronary heart disease: Impact of digital platform monitoring in preventing major cardiovascular events in Tunisia: Study protocol

**DOI:** 10.1371/journal.pone.0300250

**Published:** 2024-04-18

**Authors:** Hela Ghali, Aymen El Hraiech, Hend Ben Souda, Majdi Karray, Bruno Pavy, Chekib Zedini

**Affiliations:** 1 Faculty of Medicine of Sousse, University of Sousse, Sousse, Tunisia; 2 Department of Prevention and Security of Care, Sahloul University Hospital, Sousse, Tunisia; 3 Department of Cardiology, Sahloul University Hospital, Sousse, Tunisia; 4 Family Medicine, Faculty of Medicine of Sousse, Sousse, Tunisia; 5 Faculty of Pharmacy of Monastir, University of Monastir, Monastir, Tunisia; 6 Cardiac Rehabilitation Department, Loire-Vendée-Océan Hospital Center, Machecoul, France; 7 Department of Family and Community Medicine, Faculty of Medicine of Sousse, Sousse, Tunisia; Ataturk University Faculty of Medicine, TURKEY

## Abstract

**Background:**

Faced with the increase in the number of chronic diseases with the aging of the population, and with the observation of the insufficiency of therapeutic control, a new need has emerged, that of having a patient as a partner in care.

**Methods:**

This study is a randomized controlled trial. Patients with coronary heart disease will be recruited from one clinical site and randomly assigned into two groups: the intervention group and the control group. All participants will be followed up for a total of one year (with three-time points for data collection). Patients who are assigned to the intervention group will receive therapeutic education at first. The digital platform will then allow healthcare providers to accompany them outside the hospital walls. The primary outcome is the incidence of major cardiovascular events within one year of discharge. Main secondary outcomes include changes in health behaviors, medication adherence, and quality of life score. The digital platform is a multi-professional telemonitoring platform that allows care teams to accompany the patient outside the hospital walls. It allows the collection and transmits information from the patient’s home to the therapeutic education team. All data will be secured at a certified host. The patient application provides data on compliance, adherence to physical activity (number of steps taken per day), adequate diet (weight gain, food consumed during the meal, compliance with low-salt or salt-free diet, diabetic diet), smoking cessation, as well as medication adherence. Access to educational tools (digital media) is provided to all initial program participants. These tools will be updated annually by the rehabilitation team on the recommendations. The platform also offers the possibility of organizing an individual or group remote educational session (videoconference modules allowing group and individual sessions), a secure integrated caregiver-patient messaging system. The control group will receive the usual controls at the hospital.

**Discussion:**

To offer a complete solution of care to our patients, we have thought of setting up a digital platform that aims to monitor the patient and strengthen their abilities to manage their condition daily. This pilot experience could be generalized to several services and disciplines. It could be used in several research works.

**Trial registration:**

Trial registered with the Pan African Clinical Trial Registry (PACTR202307694422939). URL: https://pactr.samrc.ac.za/TrialDisplay.aspx?TrialID=24247.

## Introduction

Cardiovascular disease remains a leading cause of death worldwide including in Tunisia, with about 49% of the deaths from coronary heart disease (CHD) [1, 2].

Faced with the increase in the number of chronic diseases with the aging of the population, and with the observation of the insufficiency of therapeutic control, a new need has emerged, that of having a patient as a partner in care.

Participatory medicine involves a collaborative approach between patients and healthcare professionals, where patients are actively engaged in making decisions about their own treatment. This fosters better mutual understanding and enables more precise customization of medical care.

Therapeutic patient education (TPE) is a humanistic approach centered on the patient, his needs, and resources. It is proposed not only to help the patient understand the disease and treatment but also to help them become autonomous.

In fact, TPE is an approach to facilitate patient and family learning about the treatment of disease and the adoption of self-management behaviors and lifestyles to improve physical and psychosocial health outcomes (eg, biomarkers, quality of life) [3]. The goal of TPE is to improve health outcomes, including by preventing avoidable complications [4]. In prior studies, investigators have reported the positive impact of TPE on knowledge, behavioral, psychosocial, and health outcomes [5].

The appropriate application of the TPE program should result in a better quality of life for people with coronary heart disease. However, the success of these measures depends largely on the skills of patients in the daily management of their condition. Patients with coronary heart disease are often not adequately supported to develop skills in the day-to-day management of their health condition [6].

However, in most TPE programs, patient follow-up stops after the end of the education program. It is essential to assist patients with coronary heart disease in the third phase of their management.

In fact, addressing cardiovascular health in disease management was associated with a 68% and 45% reduction of incident major cardiovascular events and coronary heart disease mortality, respectively [7–9]. However, a low prevalence of ideal cardiovascular health was identified among patients with coronary heart disease, despite interventions in facilitating cardiovascular health have been developed and implemented [10–13].

Thus, our study aims to create a digital platform allowing the follow-up of patients after having benefited from a therapeutic patient education program.

This is an innovative project in our Tunisian context that offers new modes of monitoring and therapeutic support to patients suffering from cardiovascular pathology. Indeed, it is the 1st digital platform for monitoring patients with coronary heart disease in Tunisia.

This digital tool represents an innovation both for patients, who are offered a personalized monitoring and learning program tailored to their profile and wishes; and for healthcare professionals, who now have at their disposal a tool for monitoring patient objectives and modules for learning new skills.

This pilot experiment could be extended to several departments and disciplines.

This study protocol is aimed to describe the implementational plan for a controlled clinical trial in which the effect of the digital platform on major cardiovascular events reduction for patients with coronary heart disease will be evaluated. Meanwhile, the study plan for testing the effects of the TPE on facilitating adherence to recommended health behaviors (including healthy diet, regular physical activity, and smoking cessation) and medications will also be described.

## Materials and methods

### Study design

This study is a controlled clinical trial. The trial has been registered at the Pan African Clinical Trial Registry (PACTR202307694422939) after enrolment of the first participant.

The results will be reported according to the CONSORT guidelines [14]. This study is approved by the Institutional Review Committee of Sahloul University Hospital (Approval No. HS12-2022). Written informed consent will be obtained from all patients who meet the inclusion criteria and are willing to participate before randomization.

### Settings and participants

**Fig 1:**
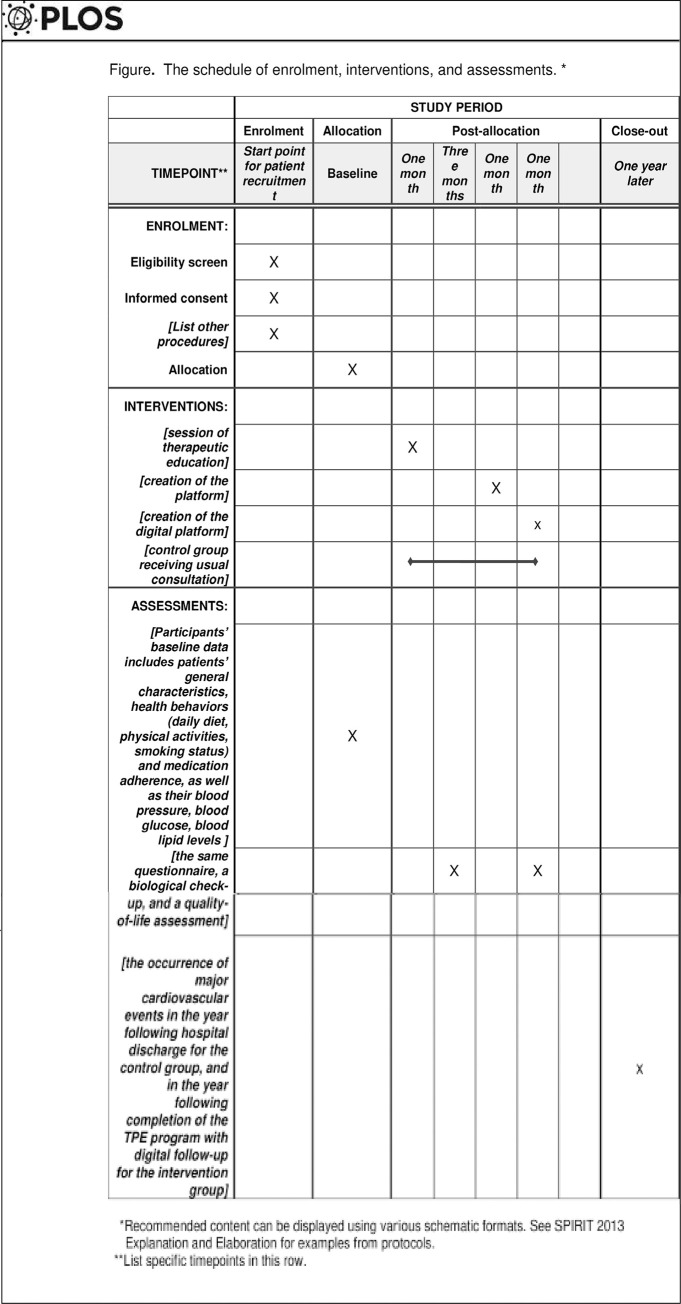
The schedule of enrolment, interventions, and assessments *. *Recommended content can be displayed using various schematic formats. See SPIRIT 2013 Explanation and Elaboration for examples from protocols. **List specific timepoints in this row.

Eligible patients will be recruited from the department of cardiology of Sahloul university hospital over one month. All participants will be followed up for a total of one year (with three time-points for data collection) (Fig 1).

Patients will be included in the study if they: (a) are 18 years or older; (b) have a documented diagnosis of coronary heart disease including acute myocardial infarction (AMI) (Non ST and ST elevation MI), patients with unstable angina, and patients with stable angina; (c) have access to a computer, and (d) agree to participate.

The exclusion criteria include: patients who are (1) with a history of psychiatric and neurological disorders; (2) unable to speak or understand Arabic; (3) with impaired bilateral hearing, or visual impairment which limits the use of computers; (4) with contraindications or severe physical disabilities that limit patients from participation; Additional exclusion criteria will be applied if patients are not able to understand the procedure and the aims of the study after full explanation.

By using the results of a meta-analysis published in 2017 [5] for an incidence of a major cardiovascular event of 13.8% in the intervention group and 38.6% in the control group, with a power of 80% and a first-species risk α of 0.05, we found a size of 56 patients in each group.

All investigators in this study will attend rigorous training on data collection and research protocol.

Training sessions will be thorough, and detailed. The purpose of this rigorous training is to ensure that all investigators have a comprehensive understanding of the data collection methods and research protocols relevant to the study.

The research protocol encompasses the set of guidelines, procedures, and ethical standards that govern how the research study will be conducted. It includes considerations such as participant recruitment, informed consent, data handling, confidentiality, and data analysis methods.

These sessions will be held before the start of the survey to prevent measurement bias. In addition, all investigators are trained in the principles of therapeutic education.

### Intervention

To identify eligible patients, a targeted review of patients’ medical record based on the inclusion and exclusive criteria will be carried out, with referrals from nurses or clinicians within the study site. During the formal screening session, we will ask patients to complete an initial screening assessment which includes age, educational background, diagnosis, heart function grade, etc. Patients who meet the study criteria will be asked to sign a written informed consent before attending the baseline visit. Participants’ baseline data includes patients’ general characteristics, health behaviors (daily diet, physical activities, smoking status) and medication adherence, as well as their blood pressure, blood glucose, blood lipid levels during hospitalization.

A total of 112 patients with coronary heart disease will be recruited at a clinical site and divided equally into an intervention group and a control group. All participants will be followed up for a total of one year (with three time-points for data collection).

### Intervention group

Patients assigned to the intervention group will first receive therapeutic education. The digital platform will then enable care providers to support them remotely.

A questionnaire will be distributed to patients before they begin the TPE program, in order to assess their knowledge of the disease, their eating habits, their physical activity, etc.

The same questionnaire will be distributed again at the end of the intervention, to study the impact of the TPE program in terms of knowledge retention.

Participants assigned to the intervention group will be divided into sub-groups. Each group will receive three TPE sessions. Educational sessions will be held once every week.

TPE sessions will include theoretical presentations on the pathophysiology of coronary artery disease, its risk factors, therapeutic objectives and therapeutic management.

These sessions will also include practical workshops using video sequences, role-playing and card games, covering heart attack alert, biological and blood pressure therapeutic objectives, the Mediterranean diet, the different commercial presentations of drugs, and types of social coverage.

At the end of each session, a round-table discussion will be held on the daily life of a coronary sufferer: physical activity/stress management/smoking/returning to work.

During the last session, each patient will benefit from an individual interview during which a lipid and glycemic assessment will be requested to evaluate the impact of the TPE in the medium term.

In addition, each patient will receive leaflets in Arabic, summarizing what will be presented during the TPE sessions.

At 3 months after completion of the TPE program, the same questionnaire will be redistributed, a biological check-up will be requested, and a quality-of-life assessment will be carried out.

In a second phase, the intervention group will be monitored via a digital platform.

### The therapeutic digital platform

The mobile application will be built using the Progressive Web App (PWA) approach. The frontend will be developed using the React framework, while the backend will be powered by Node.js. The application will also offer offline functionality, allowing users to interact with it even without an internet connection. Regarding the patient-entered data, it will be secured using the AES protocol (Advanced Encryption Standard). AES is a widely used symmetric encryption algorithm that ensures data confidentiality through its block cipher design. It operates by dividing data into fixed-size blocks and encrypting/decrypting them using a secret key. This protocol involves multiple rounds of substitution, permutation, and mixing, making it highly secure against various cryptographic attacks.

Regarding data analysis, the medical group will have access to a Power BI dashboard designed to provide real-time visualization of their patients’ information. This dashboard will include an alert module that is developed in collaboration with the medical team. The purpose of this module is to establish thresholds and alarming associations for their patients’ data, enabling timely alerts when certain conditions are met.

The application will be divided into two sections. The first section is "Patient data collection," where patients will submit their information daily.

The patient data from will be transmitted daily and visualized by the treatment team.

The second functionality is the "Education" feature, which entails sending patients notifications regarding both group and individual sessions.

This section serves not only as an information resource but also offers a secure messaging module enabling patients to interact with their healthcare team.

As for the data collected through the "Patient Information" module, it will be sent to the treatment team on a daily basis, allowing them to regularly monitor and analyze the patient’s progress.

Access to educational tools (digital media) is available to all initial program members. These tools will be updated annually by the rehabilitation team in line with the recommendations of learned societies.

The platform also offers the possibility of organizing an individual or group remote educational session (videoconferencing modules enabling group and individual sessions), and a secure integrated caregiver-patient messaging system.

*First step: Inclusion in the therapeutic digital platform*

At the end of the TPE program, the medical team verifies eligibility for the digital platform program. A "therapeutic support" contract is signed by the program participant and the medical team, setting out the therapeutic objectives during follow-up, as well as the parameters to be collected and transmitted to the platform.

The TPE team will educate the patient in the use of the mobile application, which is based on the codes and uses of consumer applications.

*Second step: digital monitoring phase*

Every day, the patient will be invited to transmit the monitoring parameters set out in the "therapeutic support" contract.

Data will be transmitted asynchronously and securely on the monitoring platform.

*Third step: Data analysis phase*

The medical team can interact with the patient via the platform. If necessary, the patient can be called into the center by the medical team for reassessment and early consultation, in order to optimize his or her care pathway.

*Forth step: Assessment phase*

At 12 months, an assessment of knowledge retention will be carried out, as well as the incidence of major cardiovascular events.

### Control group

The control group will receive the usual controls at the hospital. Patients of the control group will not receive the TPE program neither a digital monitoring.

### Outcomes

#### Primary outcome

The primary outcome of this study is the occurrence of major cardiovascular events in the year following hospital discharge for the control group, and in the year following completion of the TPE program with digital follow-up for the intervention group.

The primary outcome is based on the occurrence of acute myocardial infarction, stable angina, coronary revascularization, stroke, hospitalized heart failure, or death attributable to any cardiovascular disease [15]. Information on the primary outcome will be systematically obtained through follow-up interview at 12 months and confirmed by reviewing patients’ medical records. If a patient dies during follow-up, this information will be obtained directly from patient’s family or confirmed by physician.

#### Secondary outcomes

The secondary outcomes of the study include changes in health behaviors (smoking cessation, adoption of a healthy diet, physical activity), medication adherence, cardiovascular health score and health-related quality of life which will be assessed using the validated Tunisian version of the SF-12 health survey [16].

### Variable definitions

The American Heart Association defines the ideal cardiovascular health as simultaneous presence of four ideal health behaviors and three ideal metabolic indicators [17]. The four ideal health behaviors include nonsmoking, body mass index (BMI) < 25 kg/m2, physical activity at targeted level, and dietary consumption being consistent with current recommended guidelines. The three ideal metabolic indicators are identified as clinical parameters for blood pressure < 120/80 mm Hg, total cholesterol < 5.17 mmol/L (200 mg/dL) and fasting glucose < 5.60 mmol/L (100 mg/dL). Each component of the cardiovascular health metrics will be dichotomized as 1 (ideal status) or 0 (intermediate or poor status).

The validated Tunisian version of the SF-12 health survey (16) produces two summary scores: the Physical Component Summary (PCS) and the Mental Component Summary (MCS). These scores are standardized to have a mean of 50 in the general population, with a standard deviation of 10. Higher scores indicate better health-related quality of life.

### Data analyses

Data will be analyzed using IBM SPSS version 22.0 statistical software (SPSS Inc., Chicago, IL, USA).

Normal distribution and equality of variance will be assessed before any statistical analysis using the Kolmogorov-Smirnov test.

A descriptive analysis will be performed with mean, standard deviation, minimum and maximum values. For variables with a non-normal distribution, the median will be used as a measure of centralization, and the 25 and 75 percentiles as measures of dispersion.

Absolute and relative frequencies will be given for qualitative variables.

For quantitative data, comparisons between two means will be made using Student’s t-test or Mann-Whitney U-test. Pearson’s Chi2 test and Fisher’s exact test will be used to compare percentages.

The significance level will be set at 0.05.

### Ethical approval and consent to participate

The study protocol was approved by the Ethics Committee of Sahloul University Hospital, Sousse, Tunisia under the registration number HS12-2022, and by the Pan African Clinical Trial Registry (PACTR202307694422939).

All participants will sign a written consent form.

The investigator will present the objectives of the study and will also ensure the confidentiality and anonymity of data during analysis. Patients will be free to participate in the study.

“The authors confirm that all ongoing and related trials for this drug/intervention are registered”

## Discussion

Many studies have highlighted the impact of therapeutic education on better compliance by coronary patients, and on reducing the incidence of major cardiac events [3–5, 18–23].

However, the impact of digital patient monitoring is still poorly elucidated, especially in developing countries such as Tunisia.

In fact, a few studies carried out in developed countries have reported the benefits of using telephone applications to monitor coronary patients [24–26]. Besides, although numerous studies have been implemented with individualized mHealth-based interventions, the long-term effects of such interventions on the reductions of major cardiovascular events have not been well established. The influence of individualized mHealth based interventions on major cardiovascular events was also not determined due to small sample sizes in previous studies [25, 26].

Our work will focus on the impact of third-phase computerized follow-up of coronary patients on the incidence of major cardiac events.

In addition, the daily follow-up of these patients, together with a communication interface enabling them to report on their daily lives, will make it possible to monitor changes in their lifestyles and thus redress misbehavior prospectively.

This pilot experience could be generalized to several services and disciplines. It could be used in several research works.

### Limitations

This is a monocentric study, which leads us to discuss the generalization of the results while considering the possibility of reproducing the work in other health establishments.

In addition, using the platform requires a good internal connection. This will not be a problem, as we have good internet coverage.

Since the platform is not available in the app stores, patients currently cannot independently download and install the application. Consequently, individuals allocated to the control groups do not have access to the full functionalities offered by the platform.

## Supporting information

S1 Checklist SPIRIT 2013 checklist: Recommended items to address in a clinical trial protocol and related documents*. (DOC)
