# Supplementary material for: Therapeutic education of patients with coronary heart disease: Impact of digital platform monitoring in preventing major cardiovascular events in Tunisia: Study protocol
Source: PLoS One. 2024 Apr 18;19(4):e0300250. doi: 10.1371/journal.pone.0300250 (PMC11025886; doi:10.1371/journal.pone.0300250)
Supplement: S1 Checklist — (DOC) [file pone.0300250.s001.doc]

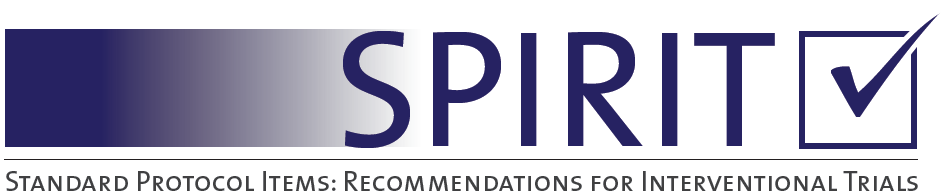


SPIRIT 2013 Checklist: Recommended items to address in a clinical trial protocol and related documents*

| Section/item | ItemNo | Description |
| --- | --- | --- |
| **Administrative information** | | |
| Title | 1 | Therapeutic education of patients with coronary heart disease: impact of digital platform monitoring in preventing major cardiovascular events in Tunisia : Study protocol |
| Trial registration | 2a | Trial registered with the Pan African Clinical Trial Registry (PACTR202307694422939). URL: https://pactr.samrc.ac.za/TrialDisplay.aspx?TrialID=24247 |
|  | 2b | All items from the World Health Organization Trial Registration Data Set |
| Protocol version | 3 | Date of approval 12/07/2023 |
| Funding | 4 | No funding received |
| Roles and responsibilities | 5a | Hela Ghali1,2*, Aymen El Hraiech1,3, Hend Ben Souda4, Majdi Karray5, Bruno Pavy6, Chekib Zedini1,7  1 University of Sousse, Faculty of Medicine of Sousse, Street of Mohamed Karoui 4000, Sousse, Tunisia.  2 Sahloul University Hospital, Department of Prevention and Security of Care, Route Ceinture Cité Sahloul 4054, Sousse, Tunisia.  3 Sahloul University hospital, Department of Cardiology, Route Ceinture Cité Sahloul 4054, Sousse, Tunisia.  4 Family Medicine, Faculty of Medicine of Sousse, Street of Mohamed Karoui 4000, Sousse, Tunisia.  5 University of Monastir, Faculty of Pharmacy of Monastir, Street Ibn Sina 5000, Monastir, Tunisia  6 Cardiac Rehabilitation Department, Loire-Vendée-Océan hospital center, 44270 Machecoul, France  7Department of Family and Community Medicine, Faculty of Medicine of Sousse, Street of Mohamed Karoui 4000, Sousse, Tunisia. |
|  | 5b | No sponsor |
|  | 5c | HG, AEH and CZ designed the trial, and provided critical revision of the manuscript. HG and AEH drafted the manuscript. HBS, MK, PB, and CZ contributed to the development of the surveys. All authors All authors reviewed and approved the final manuscript. |
|  | 5d | Composition, roles, and responsibilities of the coordinating centre, steering committee, endpoint adjudication committee, data management team, and other individuals or groups overseeing the trial, if applicable (see Item 21a for data monitoring committee) |
| Introduction |  |  |
| Background and rationale | 6a | Cardiovascular disease remains a leading cause of death worldwide including in Tunisia, with about 49% of thedeaths from coronary heart disease (CHD). Faced with the increase in the number of chronic diseases with theaging of the population, and with the observation of the insufficiency of therapeutic control, a new need hasemerged, that of having a patient as a partner in care. Therapeutic education is a humanistic approach centered on the patient, his needs, and resources. It is proposed not only to help the patient understand the disease and treatment but also to help them become autonomous. However, in most therapeutic education programs, patient follow-up stops after the end of the education program. It is essential to assist the coronary patient in the third phase of his management. Thus, our study aims to create a digital platform allowing the follow-up of patients after having benefited from a therapeutic education program. This is an innovative project in our Tunisian context that offers new modes of monitoring and therapeutic support to patients suffering from cardiovascular pathology. Indeed, it is the 1st digital platform for monitoring patients with coronary disease in Tunisia. |
|  | 6b | The control group will receive the usual controls at the hospital. Patients of the control group will not receive a therapeutic education program or a digital monitoring. |
| Objectives | 7 | This study protocol is aimed to describe the implementational plan for a controlled clinical trial in which the effect of the digital platform on major cardiovascular events reduction for patients with coronary heart disease will be evaluated. Meanwhile, the study plan for testing the effects of the TPE on facilitating adherence to recommended health behaviors (including healthy diet, regular physical activity, and smoking cessation) and medications will also be described. |
| Trial design | 8 | This study is a controlled clinical trial. It will be conducted from May 2022 to December 2023. A total of100 patients with coronary heart disease will be recruited from one clinical site and equally assigned into two groups: the intervention group and a control group. Patients who are assigned to the intervention group will receive therapeutic education at first. The digital platform will then allow healthcare providers to accompany them outside the hospital walls. The primary outcome is the incidence of major cardiovascular events within 1 year of discharge. Main secondary outcomes include changes in health behaviors, medication adherence, and quality of life score. The control group will receive the usual controls at the hospital. |
| Methods: Participants, interventions, and outcomes | | |
| Study setting | 9 | Eligible patients will be recruited from the department of cardiology of Sahloul university hospital. |
| Eligibility criteria | 10 | All participants will be followed up for a total of one year (with three time-points for data collection). Patients will be included in the study if they: (a) are 18 years or older; (b) have a documented diagnosis of coronary heart disease including acute myocardial infarction (AMI), acute coronary syndrome (ACS), or undertake a percutaneous coronary intervention (PCI) either as emergency or elective procedures; (c) have access to a computer, and (d) agree to participate.  The exclusion criteria include: patients who are (1) with a history of psychiatric and neurological disorders; (2) unable to speak or understand Arabic; (3) with impaired bilateral hearing, or visual impairment which limits the use of computers; (4) with contraindications or severe physical disabilities that limit patients from participation; Additional exclusion criteria will be applied if patients are not able to understand the procedure and the aims of the study after full explanation. |
| Interventions | 11a | To identify eligible patients, a targeted review of patients’ medical record based on the inclusion and exclusive criteria will be carried out, with referrals from nurses or clinicians within the study site. During the formal screening session, we will ask patients to complete an initial screening assessment which includes age, educational background, diagnosis, heart function grade, etc. Patients who meet the study criteria will be asked to sign a written informed consent before attending the baseline visit. Participants’ baseline data includes patients’ general characteristics, health behaviors (daily diet, physical activities, smoking status) and medication adherence, as well as their blood pressure, blood glucose, blood lipid levels during hospitalization. |
|  | 11b | A total of 100 patients with coronary heart disease will be recruited at a clinical site and divided equally into an intervention group and a control group. All participants will be followed up for a total of one year (with three time-points for data collection). |
|  | 11c | To identify eligible patients, a targeted review of patients’ medical record based on the inclusion and exclusive criteria will be carried out, with referrals from nurses or clinicians within the study site. During the formal screening session, we will ask patients to complete an initial screening assessment which includes age, educational background, diagnosis, heart function grade, etc. Patients who meet the study criteria will be asked to sign a written informed consent before attending the baseline visit. Participants’ baseline data includes patients’ general characteristics, health behaviors (daily diet, physical activities, smoking status) and medication adherence, as well as their blood pressure, blood glucose, blood lipid levels during hospitalization. |
|  | 11d | A total of 100 patients with coronary heart disease will be recruited at a clinical site and divided equally into an intervention group and a control group. All participants will be followed up for a total of one year (with three time-points for data collection). |
| Outcomes | 12 | The primary outcome of this study is the occurrence of major cardiovascular events in the year following hospital discharge for the control group, and in the year following completion of the TPE program with digital follow-up for the intervention group. The secondary outcomes of the study include changes in health behaviors (smoking cessation, adoption of a healthy diet, physical activity), medication adherence, cardiovascular health score and health-related quality of life which will be assessed using the validated Tunisian version of the SF-12 health survey |
| Participant timeline | 13 | Patients assigned to the intervention group will first receive therapeutic education. The digital platform will then enable care providers to support them remotely.  A questionnaire will be distributed to patients before they begin the TPE program, in order to assess their knowledge of the disease, their eating habits, their physical activity, etc.  The same questionnaire will be distributed again at the end of the intervention, to study the impact of the TPE program in terms of knowledge retention.  Participants assigned to the intervention group will be divided into sub-groups. Each group will receive three TPE sessions. Educational sessions will be held once every week.  TPE sessions will include theoretical presentations on the pathophysiology of coronary artery disease, its risk factors, therapeutic objectives and therapeutic management.  These sessions will also include practical workshops using video sequences, role-playing and card games, covering heart attack alert, biological and blood pressure therapeutic objectives, the Mediterranean diet, the different commercial presentations of drugs, and types of social coverage.  At the end of each session, a round-table discussion will be held on the daily life of a coronary sufferer: physical activity/stress management/smoking/returning to work.  During the last session, each patient will benefit from an individual interview during which a lipid and glycemic assessment will be requested to evaluate the impact of the TPE in the medium term.  In addition, each patient will receive leaflets in Arabic, summarizing what will be presented during the TPE sessions.  At 3 months after completion of the TPE program, the same questionnaire will be redistributed, a biological check-up will be requested, and a quality-of-life assessment will be carried out.  In a second phase, the intervention group will be monitored via a digital platform. |
| Sample size | 14 | A total of 100 patients with coronary heart disease will be recruited at a clinical site and divided equally into an intervention group and a control group. All participants will be followed up for a total of one year (with three time-points for data collection). |
| Recruitment | 15 | Strategies for achieving adequate participant enrolment to reach target sample size |
| **Methods: Assignment of interventions (for controlled trials)** | | |
| Allocation: |  |  |
| Sequence generation | 16a | Method of generating the allocation sequence (eg, computer-generated random numbers), and list of any factors for stratification. To reduce predictability of a random sequence, details of any planned restriction (eg, blocking) should be provided in a separate document that is unavailable to those who enrol participants or assign interventions |
| Allocation concealment mechanism | 16b | Mechanism of implementing the allocation sequence (eg, central telephone; sequentially numbered, opaque, sealed envelopes), describing any steps to conceal the sequence until interventions are assigned |
| Implementation | 16c | Who will generate the allocation sequence, who will enrol participants, and who will assign participants to interventions |
| Blinding (masking) | 17a | Who will be blinded after assignment to interventions (eg, trial participants, care providers, outcome assessors, data analysts), and how |
|  | 17b | If blinded, circumstances under which unblinding is permissible, and procedure for revealing a participant’s allocated intervention during the trial |
| **Methods: Data collection, management, and analysis** | | |
| Data collection methods | 18a | Plans for assessment and collection of outcome, baseline, and other trial data, including any related processes to promote data quality (eg, duplicate measurements, training of assessors) and a description of study instruments (eg, questionnaires, laboratory tests) along with their reliability and validity, if known. Reference to where data collection forms can be found, if not in the protocol |
|  | 18b | Plans to promote participant retention and complete follow-up, including list of any outcome data to be collected for participants who discontinue or deviate from intervention protocols |
| Data management | 19 | Plans for data entry, coding, security, and storage, including any related processes to promote data quality (eg, double data entry; range checks for data values). Reference to where details of data management procedures can be found, if not in the protocol |
| Statistical methods | 20a | Data will be analyzed using IBM SPSS version 22.0 statistical software (SPSS Inc., Chicago, IL, USA).  Normal distribution and equality of variance will be assessed before any statistical analysis using the Kolmogorov-Smirnov test.  A descriptive analysis will be performed with mean, standard deviation, minimum and maximum values. For variables with a non-normal distribution, the median will be used as a measure of centralization, and the 25 and 75 percentiles as measures of dispersion.  Absolute and relative frequencies will be given for qualitative variables.  For quantitative data, comparisons between two means will be made using Student's t-test or Mann-Whitney U-test. Pearson's Chi2 test and Fisher's exact test will be used to compare percentages.  The significance level will be set at 0.05. |
|  | 20b | Methods for any additional analyses (eg, subgroup and adjusted analyses) |
|  | 20c | Definition of analysis population relating to protocol non-adherence (eg, as randomised analysis), and any statistical methods to handle missing data (eg, multiple imputation) |
| **Methods: Monitoring** | | |
| Data monitoring | 21a | Composition of data monitoring committee (DMC); summary of its role and reporting structure; statement of whether it is independent from the sponsor and competing interests; and reference to where further details about its charter can be found, if not in the protocol. Alternatively, an explanation of why a DMC is not needed |
|  | 21b | Description of any interim analyses and stopping guidelines, including who will have access to these interim results and make the final decision to terminate the trial |
| Harms | 22 | Plans for collecting, assessing, reporting, and managing solicited and spontaneously reported adverse events and other unintended effects of trial interventions or trial conduct |
| Auditing | 23 | Frequency and procedures for auditing trial conduct, if any, and whether the process will be independent from investigators and the sponsor |
| Ethics and dissemination | | |
| Research ethics approval | 24 | The study protocol was approved by the Ethics Committee of Sahloul University Hospital, Sousse, Tunisia under the registration number HS12-2022, and by the Pan African Clinical Trial Registry (PACTR202307694422939).  All participants will sign a written consent form.  The investigator will present the objectives of the study and will also ensure the confidentiality and anonymity of data during analysis. Patients will be free to participate in the study. |
| Protocol amendments | 25 | Plans for communicating important protocol modifications (eg, changes to eligibility criteria, outcomes, analyses) to relevant parties (eg, investigators, REC/IRBs, trial participants, trial registries, journals, regulators) |
| Consent or assent | 26a | Who will obtain informed consent or assent from potential trial participants or authorised surrogates, and how (see Item 32) |
|  | 26b | Additional consent provisions for collection and use of participant data and biological specimens in ancillary studies, if applicable |
| Confidentiality | 27 | Data will be collected by project doctors and processed anonymously in the Prevention and Care Safety Department.  Regarding the patient-entered data, it will be secured using the AES protocol (Advanced Encryption Standard). |
| Declaration of interests | 28 | The authors declare no conflict of interest. |
| Access to data | 29 | Statement of who will have access to the final trial dataset, and disclosure of contractual agreements that limit such access for investigators |
| Ancillary and post-trial care | 30 | Provisions, if any, for ancillary and post-trial care, and for compensation to those who suffer harm from trial participation |
| Dissemination policy | 31a | Plans for investigators and sponsor to communicate trial results to participants, healthcare professionals, the public, and other relevant groups (eg, via publication, reporting in results databases, or other data sharing arrangements), including any publication restrictions |
|  | 31b | Authorship eligibility guidelines and any intended use of professional writers |
|  | 31c | Plans, if any, for granting public access to the full protocol, participant-level dataset, and statistical code |
| Appendices |  |  |
| Informed consent materials | 32 | Written informed consent will be obtained from all patients who meet the inclusion criteria and are willing to participate before randomization. |
| Biological specimens | 33 | Plans for collection, laboratory evaluation, and storage of biological specimens for genetic or molecular analysis in the current trial and for future use in ancillary studies, if applicable |

*It is strongly recommended that this checklist be read in conjunction with the SPIRIT 2013 Explanation & Elaboration for important clarification on the items. Amendments to the protocol should be tracked and dated. The SPIRIT checklist is copyrighted by the SPIRIT Group under the Creative Commons “[Attribution-NonCommercial-NoDerivs 3.0 Unported](http://www.creativecommons.org/licenses/by-nc-nd/3.0/)” license.
